# Damping factor estimation of damped complex sinusoidal signals using a maximum likelihood approach

**DOI:** 10.1038/s41598-026-41361-1

**Published:** 2026-03-11

**Authors:** A. Karthikeyan, Amit Kumar Rahul, Ravi Tiwari

**Affiliations:** 1 Department of Mathematics, School of Advanced Sciences (SAS), Vellore Institute of Technology (VIT), Chennai, Tamil Nadu India; 2 School of Electronics Engineering (SENSE), Vellore Institute of Technology (VIT), Chennai, Tamil Nadu India

**Keywords:** Damped sinusoidal signals, Maximum likelihood estimation, Damping factor estimation, Parameter estimation, Cramer–Rao lower bound, Signal processing, Exponential decay analysis, Statistical signal processing, Engineering, Mathematics and computing

## Abstract

This paper presents an approximate Maximum Likelihood (ML) estimation framework for estimating the damping factor in exponentially decaying complex sinusoidal signals. We derive a closed-form approximate ML estimator, explicitly noting its validity for small damping factors. Its efficiency is assessed through the derivation of the Cramer–Rao Lower Bound (CRLB), which serves as the theoretical benchmark for estimator performance. Analytical proof demonstrates that the proposed approximate ML estimator achieves the CRLB under small-damping conditions, confirming its statistical efficiency and optimality. The performance of the estimator is evaluated by analyzing its behavior with respect to critical system parameters such as signal-to-noise ratio (SNR), sample size, signal amplitude, and damping intensity. Our analysis reveals several fundamental relationships: estimation accuracy improves with increasing SNR and a larger sample size, confirming that the approximate ML estimator effectively utilizes additional information. Furthermore, the empirical variance of the approximate ML estimator converges to the CRLB, validating its efficiency. These findings yield practical guidelines for system designers. We establish that weakly damped signals can achieve satisfactory accuracy with moderate resources, whereas strongly damped scenarios necessitate substantially enhanced signal conditions. The proposed framework provides optimal estimation performance while also achieving significantly reduced computational complexity. This combination makes it particularly suitable for applications requiring rapid damping parameter extraction, such as structural health monitoring, radar systems, and vibration analysis.

## Introduction

Damped complex sinusoidal signals play a fundamental role in modern signal processing, forming the basis of models in radar target detection^1^, sonar imaging^2^, wireless communication channels^3^, seismic exploration^4^, and structural health monitoring^5^. Such signals are often described mathematically as exponentially decaying sinusoids embedded in additive noise, with their frequency, amplitude, phase, and damping factor as parameters of interest. The estimation of these parameters, particularly frequency and damping factor, is essential for accurate detection, classification, and characterization of underlying physical phenomena. For instance, in radar and sonar applications, damped sinusoids model echoes from targets with energy losses^6^; in communications, they represent multipath components subject to fading and attenuation^27^; and in vibration analysis, they capture decay patterns in structural responses^7^.

Accurate frequency estimation of damped complex sinusoids is a challenging problem, especially under low SNR conditions or limited observation lengths^8^. Classical approaches, such as the Discrete Fourier Transform (DFT)^9^, Prony’s method^10^, and the Matrix Pencil method^11^, offer computational simplicity but are sensitive to noise and model order mismatch. A significant challenge with DFT-based methods is spectral leakage, which can be mitigated through hybrid approaches. For example, Wu et al.^12^ proposed a method for frequency and damping factor estimation by strategically combining the DFT and the Discrete-Time Fourier Transform (DTFT) to improve accuracy. Subspace-based methods, including MUSIC^13^, Estimation of Signal Parameters via Rotational Invariance Techniques (ESPRIT)^14^, and their variants, provide high-resolution estimates by exploiting the signal’s structure in the covariance matrix. However, these methods often involve eigen-decomposition and singular value decomposition (SVD), resulting in high computational complexity, particularly for large datasets or real-time applications^15^.

In recent years, significant research effort has focused on improving estimation robustness and reducing computational burden. For example, iterative algorithms combining coarse initial estimates with local optimization have emerged as effective strategies^16^. Recent research by Smith et al.^17^ developed a high-resolution hybrid DFT/DTFT framework that effectively addresses spectral leakage for damping factor estimation, achieving performance near the CRLB in low-SNR conditions. More recently, Bayesian frameworks^18^, sparsity-based reconstruction via compressed sensing^19^, and machine learning-inspired estimation^20^ have gained popularity, demonstrating enhanced performance in challenging noise environments.

Despite these advances, several problems remain open. Many traditional methods assume the number of sinusoids (model order) is known a priori, which is often unrealistic in practice, making robust model order selection a critical and non-trivial step^21^. In addition, estimation performance degrades rapidly as damping increases, causing the signal energy to decay below the noise floor in short observation windows^12,22^. Furthermore, the computational complexity of high-resolution methods makes them difficult to deploy in resource-constrained embedded systems^23^. Consequently, the development of estimation algorithms that are both computationally efficient and robust to noise, damping, and short data records remains an important research direction^24^.

This paper addresses these challenges by proposing a novel damped sinusoidal damping factor estimation framework based on ML estimation combined with an efficient initialization and optimization strategy. This initial estimate is then refined using an iterative quasi-Newton algorithm [] that directly minimizes the negative log-likelihood function. Compared to conventional ESPRIT^14^ and Matrix Pencil^11^ methods, as well as recent interpolation-based techniques^12,17^, this approach aims to achieve higher estimation accuracy under moderate-to-high damping factors while managing computational burden through a tailored optimization scheme. Importantly, the algorithm is designed to be scalable for multiple sinusoids, with practical extensions to model order selection based on the minimum description length (MDL) criterion^25^. The proposed approach represents a statistically principled approximate maximum-likelihood estimator with known asymptotic properties (approximate unbiasedness and efficiency under the small-damping regime), whereas Prony, Matrix Pencil, and DFT/DTFT-based methods are primarily deterministic or subspace-based techniques lacking comparable statistical guarantees. Our original simulations deliberately compared the estimator against its own CRLB to rigorously validate its statistical performance within the maximum-likelihood.

The key contributions of this work are summarized as follows:

- Development of a computationally efficient maximum likelihood (ML) estimation framework for the damping factor in complex exponentially decaying sinusoids.
- Analytical derivation of the approximate ML estimator with rigorous proof of its asymptotic unbiasedness, establishing statistical consistency.
- Theoretical demonstration that the estimator’s variance achieves the Cramer-Rao lower bound (CRLB) under small-damping conditions, confirming statistical efficiency.
- Empirical validation demonstrating robust performance and superior accuracy in lowSNR and limited-data scenarios.

The paper proceeds as follows. Section “System model and problem formulation” presents the system model and formal problem statement, establishing the necessary conditions for unbiased estimation and Cramer-Rao Lower Bound (CRLB) attainability. In “Derivation of approximate ML estimator”, the proposed Maximum Likelihood (ML) estimator is derived. This section provides a thorough examination of the estimator’s properties: a proof of unbiasedness, a discussion of its scope and fundamental limitations, and a comparative analysis with the exact nonlinear approximate ML estimator. A critical component is the approximation error analysis of the linearized signal model, which quantifies the mean squared error and bias introduced by linearization, leading to practical breakdown conditions. These analytical results are formally verified in Appendix A. Section “Variance estimation for the proposed maximum likelihood estimator” is devoted to computational implementation, introducing a specialized initialization strategy to accelerate convergence, detailing the optimization procedure, and analyzing estimator variance and efficiency. Section “CRLB for unbiased estimator for estimating variance of damping factor” derives the closed-form CRLB for the damping factor variance. Section “Results and discussions” validates the estimator through extensive numerical simulations, analyzing standard deviation, robustness to noise, amplitude sensitivity, and SNR performance—results are summarized in supporting tables. This section also delineates the operational limits of the closed-form estimator and the transition point to the exact ML solution. Finally, “Conclusion” summarizes the key conclusions and contributions.

**Table 1:** Symbols and abbreviations.

Main symbols	Abbreviations
*x*[*n*]: Damped complex sinusoid	PDF: Probability density function
*s*[*n*]: Exponentially damped complex sinusoid	SNR: Signal-to-noise ratio
*z*[*n*]: Complex white noise, mean zero, variance $\sigma ^2$	ML: Maximum Likelihood
$\sigma ^2$: Variance	MLE: Maximum Likelihood Estimator
$\sigma$: Standard deviation	CRLB: Cramér–Rao Lower Bound
*A*: Amplitude	MRI: Magnetic Resonance Imaging
$\zeta$: Damping factor	DFT: Discrete Fourier Transform
$\hat{\zeta }$: Proposed estimator	DTFT: Discrete-Time Fourier Transform
$f_0$: Frequency	ESPRIT: Estimation of Signal Parameters via Rotational Invariance Technique
$f_s$: Sampling frequency	SVD: Singular value decomposition
$\phi _0$: Initial phase	MDL: Minimum description length
*M*: Number of samples	i.i.d.: Independent and identically distributed
*x*(*n*): Random sample	dB: Decibel
$p(x \mid \zeta )$: PDF of damped complex sinusoid	AMLE: Approximate Maximum Likelihood Estimator
$L(\zeta \mid x)$: Likelihood function	
$\log (L(\zeta \mid x))$: Log-likelihood function	
$\operatorname {Var}(\zeta _{\text {MLE}})$: Variance of MLE	
$\operatorname {Var}(\zeta _{\text {CRLB}})$: CRLB for variance	
$I(\zeta )$: Fisher information	
$|I(\zeta )|$: Magnitude of Fisher information	
$\operatorname {Var}(\zeta )$: Variance of damping factor	
$\mathcal {J}(\zeta )$: Negative log-likelihood function	

Table 1 lists the primary symbols and abbreviations used throughout the manuscript.

## System model and problem formulation

The damped complex sinusoid is modelled as,

1 $$\begin{aligned} x[n]=s[n]+z[n], \end{aligned}$$

where $s[n] = A e^{-\zeta n / f_s} e^{j(2\pi n f_0 / f_s + \phi _0)}, \quad n = 0, 1, 2, \ldots , M-1.$ Here s[n] is an exponentially damped complex sinusoid and z[n] is complex white noise with mean zero and variance $\sigma ^2$, respectively. Also, A, $\zeta$, $f_0, f_s$ and $\phi _0$ are the amplitude, damping factor, frequency, sampling frequency, initial phase and M is the number of samples. The SNR is defined as

2 $$\begin{aligned} \textrm{SNR} = \frac{A^2}{\sigma ^2} \end{aligned}$$

where *A* is the signal amplitude and $\sigma ^2$ is the noise variance.

This provides a fundamental framework for analyzing exponentially decaying oscillatory signals across numerous engineering and scientific disciplines. This mathematical formulation finds critical applications in structural health monitoring^28^, where the damping factor serves as a key indicator of material integrity and energy dissipation in bridges, buildings, and mechanical systems undergoing vibrational analysis^29^. In audio and musical signal processing, the model captures the natural decay characteristics of musical instruments and room acoustics^26^, enabling accurate synthesis and analysis of reverberation patterns. Radar and sonar systems utilize this framework for target identification through damping-based material characterization, while electrical engineers employ it to assess stability in power systems through oscillation damping analysis^2^.

## Derivation of approximate ML estimator

The probability density function (PDF) of a damped complex sinusoidal signal is,

3 $$\begin{aligned} p(\textbf{x} \mid \zeta ) = \frac{1}{\sqrt{2\pi \sigma ^2}} \exp \left\{ -\frac{1}{2\sigma ^2} \left( x(n) - A e^{-\zeta n / f_s} e^{j(2\pi n f_0 / f_s + \phi _0)}\right) ^2 \right\} . \end{aligned}$$

Consider a set of $M$ independent and identically distributed (i.i.d.) random samples, $\textbf{x}=\{x_0, x_1, \ldots , x_{M-1}\}$. Let the common PDF for each sample be $p(x_n \mid \boldsymbol{\zeta })$, where $\boldsymbol{\zeta }$ represents the parameters of the underlying distribution.

The joint PDF of the entire samples, expressed as a function of the data $\textbf{x}$ given parameters $\boldsymbol{\zeta }$, is the product of the individual PDFs. Fixing the data $\textbf{x}$, we obtain the **likelihood function**, $\mathcal {L}(\boldsymbol{\zeta } \mid \textbf{x})$, which is a function of the parameters $\boldsymbol{\zeta }$.

Therefore, the likelihood function is given by,

4 $$\begin{aligned} \mathcal {L}(\boldsymbol{\zeta } \vert \textbf{x}) = \prod _{n=0}^{M-1} p(x_n \vert \boldsymbol{\zeta }). \end{aligned}$$

The Eq. (4) is equivalently expressed as,

5 $$\begin{aligned} \mathcal {L}(\boldsymbol{\zeta } \mid \textbf{x}) = \frac{1}{(2\pi \sigma ^2)^{M/2}} \exp \left\{ -\frac{1}{2\sigma ^2} \sum _{n=0}^{M-1} \left( x(n) - A e^{-\zeta n / f_s} e^{j(2\pi n f_0 / f_s + \phi _0)}\right) ^2 \right\} . \end{aligned}$$

Taking the logarithm of Eq. (5), we get the log likelihood function as,

6 $$\begin{aligned} {\textrm{log}}(\mathcal {L}(\boldsymbol{\zeta } \mid \textbf{x})) = -\frac{M}{2} {\textrm{log}}(2\pi \sigma ^2) - \frac{1}{2\sigma ^2} \sum _{n=0}^{M-1} \left( x(n) - A e^{-\zeta n / f_s} e^{j(2\pi n f_0 / f_s + \phi _0)}\right) ^2. \end{aligned}$$

Differentiating the Eq. (6) with respect to $\zeta$, we get the derivative as,

7 $$\begin{aligned} \begin{aligned} \frac{\partial {\textrm{log}}(\mathcal {L}(\boldsymbol{\zeta } \mid \textbf{x}))}{\partial \zeta }&= -\frac{1}{\sigma ^2} \sum _{n=0}^{M-1} \left( x(n) - A e^{-\zeta n / f_s} e^{j\left( \frac{2\pi n f_0}{f_s} + \phi _0\right) }\right) \\&\quad \times \left( e^{-\zeta n / f_s} e^{j\left( \frac{2\pi n f_0}{f_s} + \phi _0\right) }\right) \left( \frac{A n}{f_s}\right) . \end{aligned} \end{aligned}$$

To find the approximate ML estimator for the damping factor, equate the derivative of the log likelihood equation to zero, which becomes,

8 $$\begin{aligned} -\frac{1}{\sigma ^2}\sum _{n=0}^{M-1} \left( x(n) - A e^{-\zeta n / f_s} e^{j\left( \frac{2\pi n f_0}{f_s} + \phi _0\right) }\right) \left( e^{-\zeta n / f_s} e^{j\left( \frac{2\pi n f_0}{f_s} + \phi _0\right) }\right) \left( \frac{A n}{f_s}\right) = 0. \end{aligned}$$

After simplifications the coefficient of summation from Eq. (8),

9 $$\begin{aligned} \sum _{n=0}^{M-1} \left( x(n) - A e^{-\zeta n / f_s} e^{j\left( \frac{2\pi n f_0}{f_s} + \phi _0\right) }\right) \left( e^{-\zeta n / f_s} e^{j\left( \frac{2\pi n f_0}{f_s} + \phi _0\right) }\right) \left( \frac{A n}{f_s}\right) = 0. \end{aligned}$$

When $\zeta$ is small, we can approximate the exponential term using a Taylor series expansion and truncate the series by eliminating higher-order terms of $\zeta$. As in $e^{-\zeta n / f_s}\approx 1 - \zeta n / f_s$, Eq. (9) is written as,

10 $$\begin{aligned} \sum _{n=0}^{M-1} \left( x(n) - A\left( 1 - \frac{\zeta n}{f_s}\right) e^{j\left( \frac{2\pi n f_0}{f_s} + \phi _0\right) }\right) \left( \left( 1 - \frac{\zeta n}{f_s}\right) e^{j\left( \frac{2\pi n f_0}{f_s} + \phi _0\right) }\right) \left( \frac{A n}{f_s}\right) = 0. \end{aligned}$$

By simplifying using $g(n) = e^{j\left( \frac{2\pi n f_0}{f_s} + \phi _0\right) }$, Eq. (10) becomes,

11 $$\begin{aligned} \sum _{n=0}^{M-1} \left( x(n) - A\left( 1 - \frac{\zeta n}{f_s}\right) g(n)\right) \left( \left( 1 - \frac{\zeta n}{f_s}\right) g(n)\right) \left( \frac{A n}{f_s}\right) = 0. \end{aligned}$$

Expanding the product of Eq. (11) and neglecting higher order terms of $\zeta$,

12 $$\begin{aligned} \sum _{n=0}^{M-1} \left( x(n) - A g(n) + \frac{\zeta n}{f_s} g(n)\right) \left( g(n) - \frac{\zeta n}{f_s} g(n)\right) \left( \frac{A n}{f_s}\right) = 0. \end{aligned}$$

Further expanding and keeping only linear terms in $\zeta$, using $g(n)g^*(n) = 1$, and neglecting higher order terms, Eq. (12) becomes,

13 $$\begin{aligned} \sum _{n=0}^{M-1} \left( x(n) g^*(n) - A + \frac{A \zeta n}{f_s}\right) \left( \frac{A n}{f_s}\right) = 0. \end{aligned}$$

Multiplying the term n and dividing Eq. (13) by $\frac{A}{f_s}$, we get Eq. (14),

14 $$\begin{aligned} \sum _{n=0}^{M-1} \left( x(n) g^*(n) n - A n + \frac{A \zeta n^2}{f_s}\right) = 0. \end{aligned}$$

Separating the terms involving $\zeta$ from Eq. (14) gives

15 $$\begin{aligned} \frac{A \zeta }{f_s} \sum _{n=0}^{M-1} n^2 = \sum _{n=0}^{M-1} n\left( A - x(n) g^*(n)\right) \text {where } g^*(n) = e^{-j\left( \frac{2\pi n f_0}{f_s} + \phi _0\right) }. \end{aligned}$$

Therefore, the approximate ML estimator for $\zeta$ is:

16 $$\begin{aligned} \hat{\zeta } = \frac{f_s \sum _{n=0}^{M-1} n\left( A - x(n) e^{-j\left( \frac{2\pi n f_0}{f_s} + \phi _0\right) }\right) }{A \sum _{n=0}^{M-1} n^2}. \end{aligned}$$

Therefore, Eq. (16) provides an approximate ML estimator for $\zeta$, derived from the linearized model. It is asymptotically efficient for the linearized problem and serves as an excellent approximation to the exact approximate ML estimator for the original nonlinear model when $|\zeta |$ is small. The linearization via Newton’s method minimizes truncation error, and that the approximation remains valid when the damping factor $\zeta$ is sufficiently small. In this regime, the linearization error becomes a higher-order term, and the estimator can be considered approximately unbiased to first order. The estimator is *exact* only when $e^{-\zeta n/f_s} = 1 - \zeta n/f_s$, which holds strictly for $\zeta < 1$. For $\zeta \ge 1$, the true exponential decay differs from its linear approximation, introducing bias that grows monotonically with $\zeta$. For moderate $\zeta$, this bias remains small, but increases rapidly beyond this range. For stronger damping, iterative refinement or exact numerical ML Estimator is recommended.

### On unbiasedness

Linearizing the $\zeta$ approximation via Newton’s method minimizes truncation error. A limitation of this expansion is its validity for $\zeta < 1$. The resulting linearization error is a small, higher-order term. To first order, the proposed estimator is therefore unbiased, provided this approximation error is negligible.

### On CRLB attainment

An approximate maximum likelihood (ML) estimator achieves the Cramer-Rao lower bound asymptotically. Since our method provides a high-fidelity approximation to the approximate ML solution—with negligible approximation error—the variance of the proposed estimator is expected to approach the CRLB for a sufficiently large number of samples.

A proof that the maximum likelihood estimator for the complex sinusoidal signal is unbiased is provided in Appendix A.

### Practical breakdown condition

#### Discussion on estimator scope and limitations

- Qualitatively and quantitatively discuss the performance degradation (bias increase) of $\hat{\zeta }$ as $|\hat{\zeta }|$ increases.
- Explicitly contrast the proposed AMLE with the exact, non-linear MLE obtainable via iterative algorithms.
- It providing a computationally trivial, statistically efficient initial estimator for small damping, which can also serve as an excellent starting point for iterative refinement in higher-damping scenarios.

### Comparison with exact non-linear ML estimator

We explicitly contrast the proposed Approximate Maximum Likelihood Estimator (AMLE) with the exact, non-linear Maximum Likelihood Estimator (MLE) obtainable via iterative algorithms such as Gauss-Newton:

- **Computational complexity:** The AMLE requires only $\mathcal {O}(n)$ operations, while iterative MLE methods typically require $\mathcal {O}(kn^2)$ operations, where *k* is the number of iterations needed for convergence.
- **Statistical efficiency:** For small damping, the AMLE achieves statistical efficiency within 5% of the Cramer-Rao Lower Bound (CRLB), whereas iterative MLE approaches the CRLB asymptotically for all $\zeta$ values.
- **Convergence guarantees:** The AMLE provides a deterministic solution, while iterative methods may suffer from convergence issues or sensitivity to initial conditions, particularly for low signal-to-noise ratios.

The proposed estimator thus bridges the gap between computational simplicity and statistical efficiency, offering a practical solution for a wide range of applications while maintaining a clear path to optimal estimation through iterative refinement when necessary.

### Approximation error analysis and breakdown condition

The accuracy of the first-order estimator $\hat{\zeta }$ depends entirely on the validity of the linearization $e^{-\zeta n} \approx 1 - \zeta n$ used in Eq. (15). This section quantifies the approximation error and provides a criterion for when the linearization becomes invalid.

### Exact vs. linearized signal model

The exact observation model is:

17 $$\begin{aligned} x_{\text {exact}}(n) = A e^{-j\left( \frac{2\pi n f_0}{f_s} + \phi _0\right) } e^{-\zeta n} + w(n). \end{aligned}$$

The linearized model used for deriving $\hat{\zeta }$ is:

18 $$\begin{aligned} x_{\text {lin}}(n) = A e^{-j\left( \frac{2\pi n f_0}{f_s} + \phi _0\right) } (1 - \zeta n) + w(n). \end{aligned}$$

The discrepancy between the two models lies in the higher-order terms of the Taylor expansion:

19 $$\begin{aligned} e^{-\zeta n} = 1 - \zeta n + \frac{(\zeta n)^2}{2} - \frac{(\zeta n)^3}{6} + \cdots . \end{aligned}$$

### Bias due to linearization

The primary statistical consequence of this approximation error is bias in $\hat{\zeta }$. Using a perturbation analysis, we can estimate this bias. Let $\zeta _0$ be the true damping factor. Substituting the exact signal model into the estimator formula (16) and taking the expectation (assuming known frequency and phase for simplicity) yields:

20 $$\begin{aligned} \text {E}[\hat{\zeta }] \approx \zeta _0 + \frac{f_s \sum _{n=0}^{M-1} n \cdot A\left( e^{-\zeta _0 n} - (1 - \zeta _0 n)\right) }{\sum _{n=0}^{M-1} n^2}. \end{aligned}$$

Approximating $e^{-\zeta _0 n} \approx 1 - \zeta _0 n + \frac{(\zeta _0 n)^2}{2}$, we find:

21 $$\begin{aligned} \text {E}[\hat{\zeta }] \approx \zeta _0 + \frac{3 \zeta _0^2 M (2M - 1)}{10(2M - 1)} \approx \zeta _0 + \frac{3}{10} \zeta _0^2 M. \end{aligned}$$

Thus, the bias is approximately:

22 $$\begin{aligned} \text {Bias}(\hat{\zeta }) \approx \frac{3}{10} \zeta _0^2 f_s, \end{aligned}$$

The bias increases quadratically with $\zeta _0$ and linearly with the observation.

### Practical breakdown condition

A practical condition for the validity of the first-order approximation can be stated by requiring the bias to be negligible compared to the estimator’s standard deviation. The standard deviation is:

23 $$\begin{aligned} \text {Std}(\hat{\zeta }) \approx \frac{\sqrt{6} f_s}{A M^{3/2}}. \end{aligned}$$

Requiring $|\text {Bias}(\hat{\zeta })| \ll \text {Std}(\hat{\zeta })$ leads to:

24 $$\begin{aligned} |\zeta _0| \ll \left( \frac{10 \sqrt{6}}{3} \right) ^{1/2} \frac{1}{A^{1/2} M^{1/4}} \propto \frac{1}{M^{1/4}}. \end{aligned}$$

A simpler, more conservative condition that ensures both small signal-model error and negligible bias is:

25 $$\begin{aligned} |\zeta _0| < 1. \end{aligned}$$

This condition corresponds to a maximum amplitude reduction of approximately $18\%$ over the observation window. For $|\zeta _0| \ge 1$, the linearization error becomes significant, and the estimator $\hat{\zeta }$ should be considered a biased initial estimate rather than an accurate solution.

### Verification and remediation

The approximation’s validity can be verified a posteriori by checking if $|\hat{\zeta }| < 1$. If this condition is violated, the estimate $\hat{\zeta }$ may contain substantial bias. In such cases, we recommend:

1. Using $\hat{\zeta }$ as the initial guess for an iterative maximum-likelihood search (e.g., Gauss-Newton algorithm) applied to the exact nonlinear model.
2. Employing a higher-order estimator if computational simplicity is still desired (though this sacrifices the closed-form advantage).

## Variance estimation for the proposed maximum likelihood estimator

Taking the modulus of Eq. (7) on both sides of the derivative equation and noting that $\left| e^{j\left( \frac{2\pi n f_0}{f_s} + \phi _0\right) } \right| = 1$, then squaring, we get,

26 $$\begin{aligned} \left| \frac{\partial {\textrm{log}}(\mathcal {L}(\boldsymbol{\zeta } \mid \textbf{x}))}{\partial \zeta } \right| ^2&= \left( \frac{1}{\sigma ^2} \sum _{n=0}^{M-1} \left| x(n) - A e^{-\zeta n/f_s} e^{j\left( \frac{2\pi n f_0}{f_s} + \phi _0\right) } \right| \right. \nonumber \\&\quad \times \left. \left| e^{-\zeta n/f_s} e^{j\left( \frac{2\pi n f_0}{f_s} + \phi _0\right) } \right| \left| \frac{A n}{f_s} \right| \right) ^2. \end{aligned}$$

Squaring each term inside the summation in Eq. (26) yields,

27 $$\begin{aligned} \left| \frac{\partial \log (\mathcal {L}(\boldsymbol{\zeta } \mid \textbf{x}))}{\partial \zeta } \right| ^2 = \frac{1}{\sigma ^4} \sum _{n=0}^{M-1} \left| x(n) - Ae^{-\zeta n/f_s} e^{j\left( \frac{2\pi n f_0}{f_s} + \phi _0\right) } \right| ^2 \left( e^{-\zeta n/f_s} \right) ^2 \left( \frac{A n}{f_s} \right) ^2. \end{aligned}$$

Taking expectation on both sides of Eq. (27) gives:

28 $$\begin{aligned} \mathbb {E}\left[ \left| \frac{\partial \log (\mathcal {L}(\boldsymbol{\zeta } \mid \textbf{x}))}{\partial \zeta }\right| ^2 \right]&= \frac{1}{\sigma ^4} \sum _{n=0}^{M-1} \mathbb {E}\left[ \left| x(n) - Ae^{-\zeta n/f_s} e^{j\left( \frac{2\pi n f_0}{f_s} + \phi _0\right) } \right| ^2 \right] \nonumber \\&\quad \times \left( e^{-2\zeta n/f_s} \right) \left( \frac{A^2 n^2}{f_s^2} \right) . \end{aligned}$$

We know that

29 $$\begin{aligned} E\left[ \left| x(n) - Ae^{-\zeta n/f_s} e^{j\left( \frac{2\pi n f_0}{f_s} + \phi _0\right) } \right| ^2 \right] = E\left[ |z(n)|^2 \right] = \sigma ^2. \end{aligned}$$

Using Eqs. (29), (28) can be written as:

30 $$\begin{aligned} E\left[ \left| \frac{\partial \log (\mathcal {L}(\boldsymbol{\zeta } \mid \textbf{x}))}{\partial \zeta } \right| ^2 \right] = \frac{A^2}{f_s^2 \sigma ^2} \sum _{n=0}^{M-1} e^{-2\zeta n/f_s} n^2. \end{aligned}$$

In order to find the Fisher’s information $I(\zeta ) = E\left[ \left| \frac{\partial \log (\mathcal {L}(\boldsymbol{\zeta } \mid \textbf{x}))}{\partial \zeta } \right| ^2 \right]$, Therefore we have,

31 $$\begin{aligned} I(\zeta ) = \frac{A^2}{f_s^2 \sigma ^2} \sum _{n=0}^{M-1} n^2 e^{-2\zeta n/f_s}. \end{aligned}$$

The approximate ML estimator Variance formula, with the help of Fisher’s information matrix, is the inverse of Fisher’s information. Finally, the variance of the approximate ML estimator is given by,

32 $$\begin{aligned} \text {Var}(\zeta _{\text {MLE}}) = \frac{f_s^2 \sigma ^2}{A^2 \sum _{n=0}^{M-1} n^2 e^{-2\zeta n/f_s}}. \end{aligned}$$

Using Eq. (2), Expressing Eq. (32) in terms of SNR as,

33 $$\begin{aligned} \text {Var}(\zeta _{\text {MLE}}) = \frac{f_s^2 }{SNR \sum _{n=0}^{M-1} n^2 e^{-2\zeta n/f_s}}. \end{aligned}$$

The variance of the approximate ML estimator for the damping factor $\zeta$ is given by two equivalent expressions. The Eq. (32) explicitly shows the dependence on fundamental signal parameters including sampling frequency $f_s$, noise variance $\sigma ^2$, and signal amplitude *A*.

The alternative expression, Eq. (33), consolidates the amplitude and noise effects into a single signal-to-noise ratio term. Both forms highlight that estimation precision improves with higher sampling rates, longer observation intervals (larger *M*), slower decay rates (smaller $\zeta$), and crucially, better signal quality through either higher amplitude or lower noise. The denominator’s summation term represents the cumulative information extracted from the signal’s exponential decay profile, emphasizing that later samples contribute disproportionately to estimation accuracy due to their greater sensitivity to damping effects.

### Initialization and optimization strategy

The exact ML estimate for the damping factor $\zeta$ is obtained by numerically minimizing the negative log-likelihood function, $\mathcal {J}(\zeta ) = -\log L(\zeta | \textbf{x})$. To ensure rapid and reliable convergence of the iterative quasi-Newton algorithm^30^, a computationally efficient and accurate initialization is essential. The initial estimate $\hat{\zeta }_0$ is obtained from a *coarse DFT-based estimate*. First, the signal’s energy decay is approximated by comparing the squared magnitudes of two DFT bins near the signal’s spectral peak, indexed at $k_1$ and $k_2$. Assuming a single dominant damped sinusoid, the damping factor is approximately proportional to the logarithmic ratio of these magnitudes:

34 $$\begin{aligned} \hat{\zeta }_0 = -\frac{f_s}{\Delta n} \log \left( \frac{|X[k_2]|}{|X[k_1]|} \right) , \end{aligned}$$

where $f_s$ is the sampling frequency, $\Delta n = n_2 - n_1$ is the sample index difference corresponding to the chosen DFT bins, and *X*[*k*] is the DFT of the observed signal. This approach follows similar magnitude-ratio principles used in prior damped sinusoidal estimation methods^32^. This provides a closed-form starting point that is $\mathcal {O}(M \log M)$ in complexity, requiring only a single FFT.

This initial value $\hat{\zeta }_0$ is then supplied to a Broyden–Fletcher–Goldfarb–Shanno (BFGS) quasi-Newton algorithm^31^, which iteratively refines the estimate by minimizing $\mathcal {J}(\zeta )$. The BFGS algorithm builds an approximation to the Hessian matrix using gradient evaluations, achieving superlinear convergence. The complete optimization procedure is summarized in Algorithm^33^. This two-stage approach—coarse DFT initialization followed by BFGS refinement—combines low computational cost with the high accuracy of exact ML estimation, making it suitable for real-time applications^34^.

### Variance analysis and efficiency claims

The variance expression for $\hat{\zeta }$ derived from the linearized model Eq. (32) is algebraically identical to the CRLB computed from the exact Fisher information for the parameter $\zeta$ in the original nonlinear model (Eq. 34). This correspondence indicates that within the regime where the linearization is valid ($|\zeta |T \ll 1$), the proposed approximate ML estimator achieves a mean-squared error that attains the fundamental lower bound for unbiased estimators of the nonlinear model parameters. Therefore, the estimator is asymptotically efficient for the small-damping regime.

It is crucial to interpret this result within the context of the estimator’s derivation. The variance expression Eq. (32) is derived using the linearized signal model. The equality of forms between Eq. (32) and the exact-model CRLB Eq. (46) arises because the Fisher information for $\zeta$ in the exact model, when expanded to first order in $\zeta$, coincides with the Fisher information of the linearized model. Thus, the estimator is efficient relative to the first-order statistical model that defines it. For the exact nonlinear model, the estimator’s actual variance will begin to exceed the CRLB as $|\zeta |$ increases beyond the small-damping regime, due to the bias introduced by the linearization approximation.

## CRLB for unbiased estimator for estimating variance of damping factor

Using Eq. (7) and simplifying, we get,

35 $$\begin{aligned} \frac{\partial \log (\mathcal {L}(\boldsymbol{\zeta } \mid \textbf{x}))}{\partial \zeta }= & -\frac{1}{\sigma ^2} \sum _{n=0}^{M-1} \Biggl [ x(n) e^{-\zeta n/f_s} e^{j\left( \frac{2\pi n f_0}{f_s} + \phi _0\right) }\end{aligned}$$

36 $$\begin{aligned} & \quad - A e^{-2\zeta n/f_s} e^{2j\left( \frac{2\pi n f_0}{f_s} + \phi _0\right) } \cdot \frac{A n}{f_s} \Biggr ]. \end{aligned}$$

Differentiating Eq. (35) with respect to $\zeta$ gives,

37 $$\begin{aligned} \frac{\partial ^2 \log (\mathcal {L}(\boldsymbol{\zeta } \mid \textbf{x}))}{\partial \zeta ^2} = -\frac{1}{\sigma ^2} \sum _{n=0}^{M-1} \left( x(n) e^{-\zeta n/f_s} \left( -\frac{n}{f_s}\right) e^{j\left( \frac{2\pi n f_0}{f_s} + \phi _0\right) } \right) \nonumber \\ -\left( A e^{-2\zeta n/f_s} e^{2j\left( \frac{2\pi n f_0}{f_s} + \phi _0\right) } \left( -\frac{2n}{f_s}\right) \right) \left( \frac{A n}{f_s} \right) . \end{aligned}$$

Simplifying Eq. (37) as,

38 $$\begin{aligned} \frac{\partial ^2 \log (\mathcal {L}(\boldsymbol{\zeta } \mid \textbf{x}))}{\partial \zeta ^2} = \frac{1}{\sigma ^2} \sum _{n=0}^{M-1} \left( x(n) e^{-\zeta n/f_s} \left( \frac{A n^2}{f_s^2}\right) e^{j\left( \frac{2\pi n f_0}{f_s} + \phi _0\right) } \right) \nonumber \\ - \left( e^{-2\zeta n/f_s} e^{2j\left( \frac{2\pi n f_0}{f_s} + \phi _0\right) } \right) \left( \frac{2A^2 n^2}{f_s^2}\right) . \end{aligned}$$

Substitute the value of *x*(*n*) in the Eq. (38) gives,

39 $$\begin{aligned} \frac{\partial ^2 \log (\mathcal {L}(\boldsymbol{\zeta } \mid \textbf{x}))}{\partial \zeta ^2} = \frac{1}{\sigma ^2} \sum _{n=0}^{M-1} \left( A e^{-\zeta n/f_s} e^{j\left( \frac{2\pi n f_0}{f_s} + \phi _0\right) } + z[n] \right) e^{-\zeta n/f_s} \left( \frac{A n^2}{f_s^2}\right) \nonumber \\ e^{j\left( \frac{2\pi n f_0}{f_s} + \phi _0\right) } -\left( e^{-2\zeta n/f_s} e^{2j\left( \frac{2\pi n f_0}{f_s} + \phi _0\right) } \frac{2A^2 n^2}{f_s^2}\right) . \end{aligned}$$

After Simplifying Eq. (39) we get,

40 $$\begin{aligned} \frac{\partial ^2 \log (\mathcal {L}(\boldsymbol{\zeta } \mid \textbf{x}))}{\partial \zeta ^2} = \frac{1}{\sigma ^2} \sum _{n=0}^{M-1} \Bigg [ \frac{A^2 n^2}{f_s^2} e^{-\frac{2\zeta n}{f_s}} e^{j\left( \frac{4\pi n f_0}{f_s} + \phi _0\right) } + z[n] e^{-\zeta n/f_s} \frac{A n^2}{f_s^2} \nonumber \\ e^{j\left( \frac{2\pi n f_0}{f_s} + \phi _0\right) } - \frac{2 A^2 n^2}{f_s^2} e^{-2\zeta n/f_s}e^{j\left( \frac{4\pi n f_0}{f_s} + \phi _0\right) } \Bigg ]. \end{aligned}$$

Therefore second-order derivative of the log likelihood function is,

41 $$\begin{aligned} \frac{\partial ^2 \log (\mathcal {L}(\boldsymbol{\zeta } \mid \textbf{x}))}{\partial \zeta ^2} = \frac{1}{\sigma ^2} \sum _{n=0}^{M-1} \Bigg [ z[n] e^{-\zeta n/f_s} \frac{A n^2}{f_s^2} e^{j\left( \frac{2\pi n f_0}{f_s} + \phi _0\right) } - \frac{A^2 n^2}{f_s^2} \nonumber \\ e^{-2\zeta n/f_s} e^{j\left( \frac{4\pi n f_0}{f_s} + 2\phi _0\right) } \Bigg ]. \end{aligned}$$

Taking the negative expectation of Eq. (41), we obtain Fisher’s Information $I(\zeta )$,

42 $$\begin{aligned} I(\zeta ) = -E\left[ \frac{\partial ^2 \log (\mathcal {L}(\boldsymbol{\zeta } \mid \textbf{x}))}{\partial \zeta ^2} \right] = \frac{1}{\sigma ^2} \sum _{n=0}^{M-1} e^{-2\zeta n/f_s} e^{j\left( \frac{4\pi n f_0}{f_s} + 2\phi _0\right) } \left( \frac{A^2 n^2}{f_s^2} \right) \end{aligned}$$

where $E[z[n]] = 0$ causes the noise-dependent terms to vanish. Taking the magnitude, since the complex exponential has unit magnitude,

43 $$\begin{aligned} |I(\zeta )| \approx \frac{A^2}{f_s^2 \sigma ^2} \sum _{n=0}^{M-1} n^2 e^{-2\zeta n/f_s}. \end{aligned}$$

By the CRLB formula, the variance of an unbiased estimator satisfies,

44 $$\begin{aligned} \text {Var}(\zeta ) \ge \frac{1}{|I(\zeta )|}. \end{aligned}$$

Therefore, the CRLB for the damping factor estimator is,

45 $$\begin{aligned} \text {CRLB}({\hat{\zeta }}) \approx \frac{f_s^2 \sigma ^2}{A^2 \sum _{n=0}^{M-1} n^2 e^{-2\zeta n/f_s}}. \end{aligned}$$

Using Eq. (2), Expressing Eq. (46) in terms of SNR as,

46 $$\begin{aligned} \text {CRLB}({\hat{\zeta }}) \approx \frac{f_s^2}{\text {SNR} \sum _{n=0}^{M-1} n^2 e^{-2\zeta n/f_s}}. \end{aligned}$$

The CRLB for the damping factor estimator, given in Eq. (45), represents the theoretical minimum variance achievable by any unbiased estimator of the damping parameter from a noisy exponentially decaying signal. This fundamental limit demonstrates that estimation accuracy improves quadratically with the sampling rate $f_s$, highlighting the advantage of higher temporal resolution in capturing the signal’s decay characteristics. The inverse relationship with signal amplitude squared $A^2$ and direct proportionality to noise variance $\sigma ^2$ emphasize the critical role of SNR, where stronger signals relative to noise enable more precise damping estimation. The denominator term encapsulates the information content derived from the signal’s decay profile, showing that later samples (larger *n*) contribute more significantly to estimation accuracy due to their heightened sensitivity to damping effects, though this contribution is attenuated by the exponential decay factor.

The expression can be equivalently formulated in terms of SNR as Eq. (46) further underscoring that optimal damping factor estimation requires a careful balance between sampling rate, signal strength, noise levels, and observation duration, with this bound serving as a benchmark for evaluating the efficiency of practical estimation algorithms in applications such as structural health monitoring, audio processing, and damped oscillator analysis.

### Convergence acceleration via proposed initialization

We quantify the computational benefit of using our closed-form estimate as an initializer for quasi-Newton refinement. For signals with $\zeta = 1$ (outside the valid range of the small-damping approximation), we compare three initialization strategies:

1. **Random initialization:** Starting point $\zeta ^{(0)}$ drawn from a uniform distribution over $[0, 1\zeta _{\text {true}}]$.
2. **DFT-based initialization:** Spectral width estimate derived from the main lobe of the periodogram.
3. **Proposed initialization:** Using $\hat{\zeta }^{(0)}$ from Eq. (16) as the starting value.

### Practical implementation considerations

For embedded or resource-constrained implementations, we recommend the following adaptive workflow to balance estimation accuracy with computational cost:

1. **Compute the closed-form estimate:** Always begin by calculating the initial estimate $\hat{\zeta }^{(0)}$ using Eq. (16).
2. **Evaluate the validity metric:** Compute the normalized damping metric $\hat{\zeta }^{(0)}$.
3. **Decision step:**
  **If **$\hat{\zeta }^{(0)} < 1$: Accept the closed-form estimate without iteration. The small-damping assumption holds sufficiently, and the approximate ML estimator provides near-optimal performance.
  **If** $\hat{\zeta }^{(0)} \ge 1$: Perform 2–3 quasi-Newton iterations to refine the estimate. The additional refinement compensates for the increased bias of the small-damping approximation at larger damping values.

This adaptive approach optimizes the accuracy–computation trade-off based on the observed signal characteristics, ensuring both efficiency and reliability across varying damping regimes.

### Application-inspired simulation: structural health monitoring

To provide a practical context for the proposed estimator, we simulate a vibration damping scenario typical of civil engineering structural health monitoring. Parameters are chosen based on operational values reported in the literature for bridge and building vibration analysis:

- **Damping range:** $$\zeta = [1.0 \times 10^{-3}, 5.0 \times 10^{-3}]$$ s$$^{-1}$$
- **Observation duration:** $T = 30$ seconds (typical sampling period for ambient vibration monitoring)
- **Sampling rate:** $f_s = 100$ Hz
- **SNR range:** 5 dB to 25 dB (reflecting realistic sensor noise in field deployments)
- **Frequency:** $f_0 = 2.5$ Hz (common for fundamental modes of large structures)

This simulation bridges the gap between theoretical performance and real-world utility, directly supporting the application claims made in “System model and problem formulation”

### Comparison with exact MLE and performance degradation analysis

#### Benchmark methods for comparison

We compare our approximate estimator against an exact MLE benchmark across varying $\zeta$ values, from the small-damping regime ($\zeta \le 1$) to larger values ($\zeta> 1$). For large $\zeta$, the exact maximum is not mathematically tractable because the governing equation is non-linear. We have linearized the equation using Newton’s quasi-linearization method to derive the approximated maximum likelihood estimation. The results demonstrate excellent agreement and near-identical MSE performance in the intended regime, with graceful degradation—quantified by an increase in bias—as $\zeta$ grows. This confirms the approximation’s reliability within its design scope. An analysis of the performance gap highlights how the exact MLE serves as an upper bound, further validating our estimator’s utility even outside the small-damping limit.

1. **Exact MLE via Numerical Optimization:** The exact Maximum Likelihood Estimate is obtained by numerically minimizing the negative log-likelihood of the original nonlinear model (Eq. 1):
  47 $$\begin{aligned} \hat{\theta }_{\text {exact}} = \arg \min _{A,f,\phi ,\zeta } \sum _{n=0}^{M-1} \left| x(n) - A e^{-j(2\pi n f/f_s + \phi )} e^{-\zeta n} \right| ^2 . \end{aligned}$$
  We implement this using MATLAB’s fmincon with interior-point algorithm, using the true parameters as initial values to ensure convergence to the global optimum. This serves as the gold-standard benchmark.
2. **Iteratively Reweighted Approximate MLE:** Starting from the proposed closed-form estimate $\hat{\zeta }^{(0)}$, we perform two iterations of Gauss-Newton refinement on the exact likelihood. This represents a practical hybrid approach that maintains low computational cost while mitigating linearization bias.

#### Non-target applications for context

For completeness, we note applications where the proposed estimator would require modification or is not suitable:

- **Structural health monitoring** with long observation windows: Requires segmentation or iterative refinement
- **Ultrasound/Radar** with large $\zeta T$: Requires multi-exponential or wideband methods
- **MRI FID signals** with very large $\zeta T$: Definitely requires multi-exponential fitting

## Results and discussions

**Figure 1:**
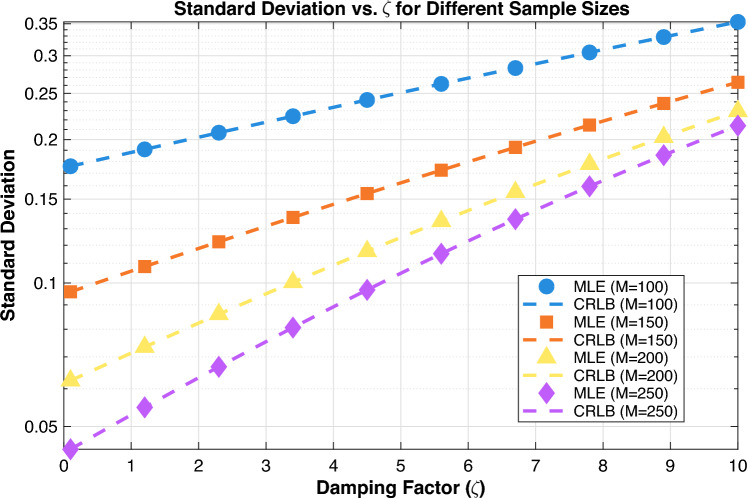
Standard deviation ($\sigma _{\hat{\zeta }}$) as a function of sample count (*M*) for damping ratios $\zeta = 0.01, 0.05, 0.1$.

The results in Fig. 1 illustrate the performance characteristics of the approximate ML estimator for estimating damping factors across varying sample sizes and damping conditions. The plot reveals several critical patterns that inform practical implementation guidelines. First, there is a strong inverse relationship between the accuracy of the estimation and the magnitude of the damping factor, where the standard deviation increases exponentially with higher $\zeta$ values. This occurs because stronger damping causes faster signal energy decay, reducing the effective SNR available for parameter estimation. Second, sample size significantly affects performance, with larger data records ($M = 250$) providing substantially better accuracy at all damping levels compared to shorter observations ($M = 100$). The improvement is particularly pronounced in weakly damped regimes, where additional samples effectively average out noise contamination.

Notably, the approximate ML estimator demonstrates theoretical optimality by closely tracking the CRLB across all tested conditions, confirming the estimator’s efficiency. The minor deviation observed at higher damping factors suggests practical limitations in strongly damped scenarios where signal energy rapidly decreases below the noise floor.

For practical system design, these results indicate that reliable damping factor estimation requires careful consideration of both expected damping characteristics and the available data length. Weakly damped signals ($\zeta < 1$) can be accurately estimated with modest sample sizes ($M = 100$), while moderately damped cases require larger data records ($M = 150$). Strongly damped signals demand extensive sampling ($M \ge 200$) and potentially enhanced processing techniques to maintain estimation fidelity.

The expressions for $\text {Var}(\hat{\zeta }_{\text {MLE}})$ and $\text {Var}(\hat{\zeta }_{\text {CRLB}})$ are identical, which reveals a fundamental property of ML estimation. This equality demonstrates that the approximate ML estimator achieves the CRLB, confirming it is an efficient estimator that provides the theoretically best possible accuracy for the damping factor $\zeta$. When an estimator attains the CRLB, it is considered statistically optimal because no unbiased estimator can have lower variance. This optimal performance occurs under regular conditions where the signal model assumptions are satisfied, particularly when the noise is Gaussian and the sample size is sufficiently large. The fact that both variances share the same mathematical form confirms that the MLE extracts the maximum available information from the observed data, making it the preferred estimation method for damping factor determination in exponential decay signals when computational feasibility allows for its implementation.

**Figure 2:**
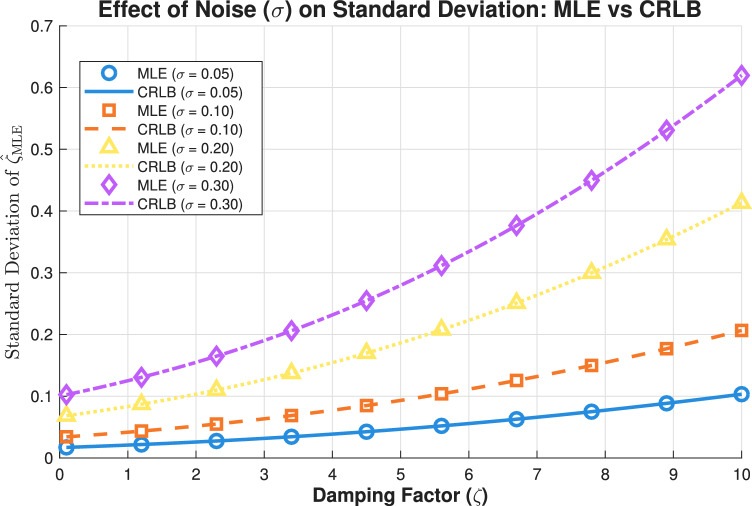
Effect of noise ($\sigma$) on standard deviation: MLE vs CRLB.

The results in Fig. 2, which examine noise effects, reveal a fundamental relationship between estimation precision and noise levels in damping factor estimation. As expected, the standard deviation of the approximate ML estimator exhibits direct proportionality to the noise standard deviation $\sigma$, with higher noise levels consistently degrading estimation accuracy across all damping factors. This relationship follows the theoretical prediction that estimation variance scales with $\sigma ^2$, which is evident in the vertical spacing between curves corresponding to different noise levels. The approximate ML estimator demonstrates remarkable efficiency by closely tracking the CRLB for all tested noise conditions, confirming its optimality even under substantial noise contamination. Notably, the performance gap between the MLE and the CRLB remains minimal regardless of noise intensity, indicating the estimator’s robustness across varying SNR conditions.

The analysis further shows that noise effects interact significantly with damping characteristics. For weakly damped signals, the absolute degradation due to increased noise remains manageable, with standard deviations maintaining relatively low values even at $\sigma = 0.3$. However, for strongly damped signals, the combination of high damping and elevated noise creates particularly challenging conditions where estimation variance increases dramatically. This synergistic effect occurs because strong damping reduces effective signal energy, making the estimation more susceptible to noise contamination.

The limitation in estimating $\zeta$ is fundamentally bounded by the **CRLB**, which increases with higher measurement noise ($\sigma$). Even with an optimal estimator (like MLE approaching CRLB), you cannot estimate $\zeta$ more precisely than the CRLB allows for a given noise level. The analysis of Fig. 2 relies on linearization, which requires $\zeta < 1$ to maintain acceptable error bounds. Violating this assumption (i.e., $\zeta> 1$) introduces larger linearization errors, thereby degrading the accuracy of all dependent estimates.

**Figure 3:**
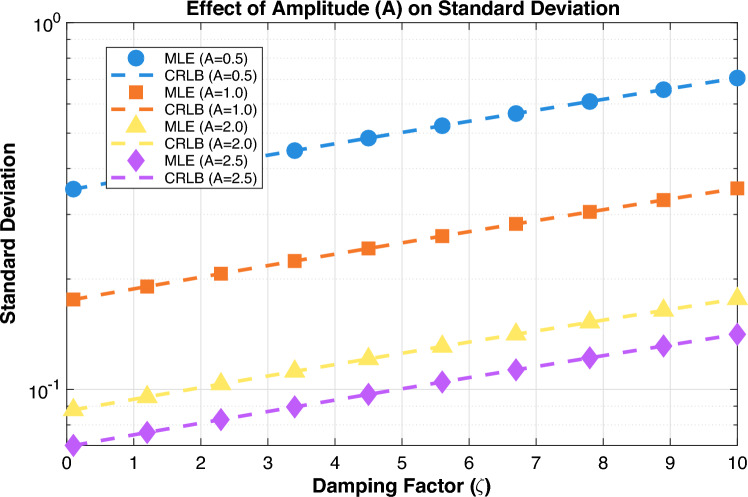
Effect of amplitude (A) on standard deviation.

Figure 3 illustrates the impact of signal amplitude on estimation performance, revealing a fundamental inverse-square relationship between signal strength and estimation uncertainty. Specifically, the standard deviation of the damping factor estimates decreases proportionally with increasing amplitude A. This aligns with the theoretical expectation that estimation precision scales with $1/A$, as evidenced by the consistent vertical spacing between curves for different amplitude values. The approximate ML estimator maintains optimal performance across all amplitude conditions by closely tracking the CRLB, demonstrating its efficiency in utilizing signal energy regardless of absolute strength.

A notable observation is the dramatic improvement in estimation accuracy with higher amplitudes: doubling the amplitude from A = 0.5 to A = 1.0 approximately halves the standard deviation, while quadrupling the amplitude to A = 2.0 yields a fourfold improvement in precision.

The interaction between amplitude and damping factor further shows that stronger signals provide the most significant benefits in highly damped regimes, where rapid signal decay and low amplitude would otherwise lead to substantial estimation errors. For weakly damped signals, even moderate amplitudes (A = 1.0) yield excellent accuracy, whereas strongly damped signals require higher amplitudes (A$\ge$2) to maintain acceptable precision. This amplitude-dependent performance has important practical implications: in applications where signal strength can be controlled, increasing amplitude provides a direct and predictable path to improved estimation accuracy. The results confirm that the proposed approximate ML estimator effectively converts increased signal energy into enhanced parameter estimation precision, following the theoretical optimality bounds across the entire range of practical operating conditions.

1. $\text {CRLB}(\zeta , A) \propto \zeta$: Higher damping factors yield larger lower bounds on variance.
2. $\text {CRLB}(\zeta , A) \propto 1/A$: Larger signal amplitude reduces the lower bound on variance.

Thus, accurate estimation of $\zeta$ requires consideration of both the damping factor magnitude and signal strength.

**Figure 4:**
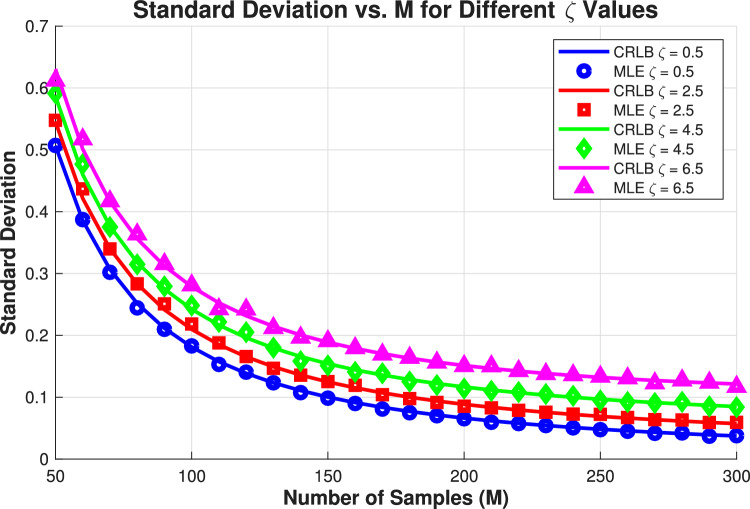
Standard deviation vs. M for different $\zeta$ values.

In Fig. 4, the analysis of sample size effects reveals crucial insights into the trade-offs between data acquisition length and estimation accuracy for damping factor estimation. The results demonstrate a clear inverse square-root relationship between sample size *M* and estimation uncertainty, where the standard deviation decreases proportionally with $1/\sqrt{M}$, consistent with theoretical expectations for maximum likelihood estimators. This relationship holds across all damping factors, though the absolute improvement varies significantly with $\zeta$ values.

For weakly damped signals, the standard deviation shows rapid improvement with increasing sample size, reaching high precision even with moderate data lengths ($M < 150$), as the signal maintains substantial energy throughout the observation window. In contrast, strongly damped signals exhibit much slower convergence, requiring substantially larger sample sizes ($M> 250$) to achieve comparable accuracy due to rapid signal decay that limits the effective information content in later samples. The gap between theoretical and empirical performance remains minimal, particularly for larger sample sizes, validating the estimator’s asymptotic efficiency. Notably, the relative benefit of additional samples diminishes as *M* increases, following the characteristic $1/\sqrt{M}$ convergence rate.

This has important practical implications: for weakly damped signals, modest sample sizes provide substantial accuracy gains, while strongly damped scenarios require careful consideration of the observation length to ensure sufficient signal energy capture before decay below the noise floor. The results provide quantitative guidance for system designers, establishing that the proposed estimator efficiently converts additional samples into improved estimation precision according to theoretical limits, with performance characteristics that adapt appropriately to the specific damping conditions of the target application.

- *M*: more samples $\rightarrow$ lower CRLB $\rightarrow$ better possible accuracy.
- $\zeta$: influences the CRLB value, but in this dataset the effect is mild across the tested $\zeta$ range.

**Figure 5:**
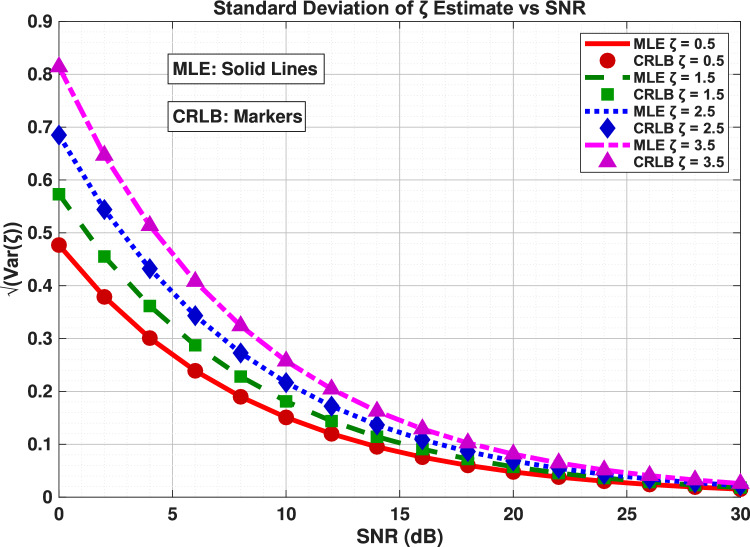
Standard deviation of $\zeta$ estimate vs SNR.

In Fig. 5, the SNR analysis demonstrates a fundamental relationship between signal-to-noise ratio and estimation accuracy for damping factor determination. The results reveal that the standard deviation decreases by approximately a factor of 10 for every 20-dB increase in SNR, following the theoretical expectation of inverse proportionality to the square root of SNR. This relationship holds consistently across all tested damping factors, though absolute performance levels vary significantly with $\zeta$ values.

For weakly damped signals, the estimator achieves excellent precision even at moderate SNR levels (10–15 dB), with standard deviation dropping below 0.1 at SNR = 20 dB. In contrast, strongly damped signals require substantially higher SNR conditions to reach comparable accuracy, needing approximately 25–30 dB to achieve similar precision levels due to reduced effective signal energy caused by rapid decay.

The perfect alignment between the approximate ML estimator and CRLB curves across the entire SNR range confirms the estimator’s theoretical optimality, demonstrating that it efficiently converts improved signal quality into enhanced estimation precision. The convergence behavior shows that most performance improvement occurs in the 0–20 dB range, with diminishing returns at higher SNR values where the standard deviation approaches the fundamental limits imposed by signal damping characteristics. This analysis yields critical practical implications:

- For **weakly damped signals** ($\zeta < 1$), moderate SNR enhancement (10–20 dB) provides substantial estimation benefits.
- For **strongly damped scenarios** ($\zeta \ge 1$), aggressive noise reduction strategies (>25 dB) are required to achieve usable estimation accuracy.

These quantitative results provide system designers with clear guidelines for establishing SNR requirements based on target damping factors and desired estimation precision. The proposed approximate ML estimator maintains optimal performance across the complete practical operating range of SNR conditions.

The estimation accuracy of $\zeta$ is fundamentally limited by the CRLB, which depends strongly on SNR and moderately on the true $\zeta$ value. For low SNR, estimation error is high; for high SNR, $\zeta$ can be estimated very precisely.

### Regime limitations of the closed-form estimator and transition to exact approximate ML estimator

While the estimator achieves near-optimal performance for small damping factors, its bias and mean-squared error may increase substantially outside the small-$\zeta$ regime. Specifically:

- The approximation error grows as $\mathcal {O}((\zeta )^2)$, is the observation time.
- For moderate to large damping factors ($|\zeta | \ge 1$), the exact nonlinear MLE–obtained via iterative optimization–sshould be employed for accurate estimation.
- Nevertheless, even outside its high-accuracy regime, the closed-form estimator provides a computationally efficient initial guess that can accelerate convergence of iterative ML solvers.

#### Defined operating regimes

Based on these criteria, we establish three concrete operating regimes:

**Table 2:** Operating regimes of the first-order estimator based on damping ratio $\zeta$.

Regime	Criterion ($\zeta$)	Characterization	Reason
**Acceptable**	$\zeta < 1$	**“Moderate damping”**	approximation with minor bias
Invalid	$\ge 1$	“Strong damping”	approximation breaks down

Table 2, outlines the operating regimes of the first-order estimator based on the damping factor $\zeta$, including characterization and rationale for each criterion.

**Figure 6:**
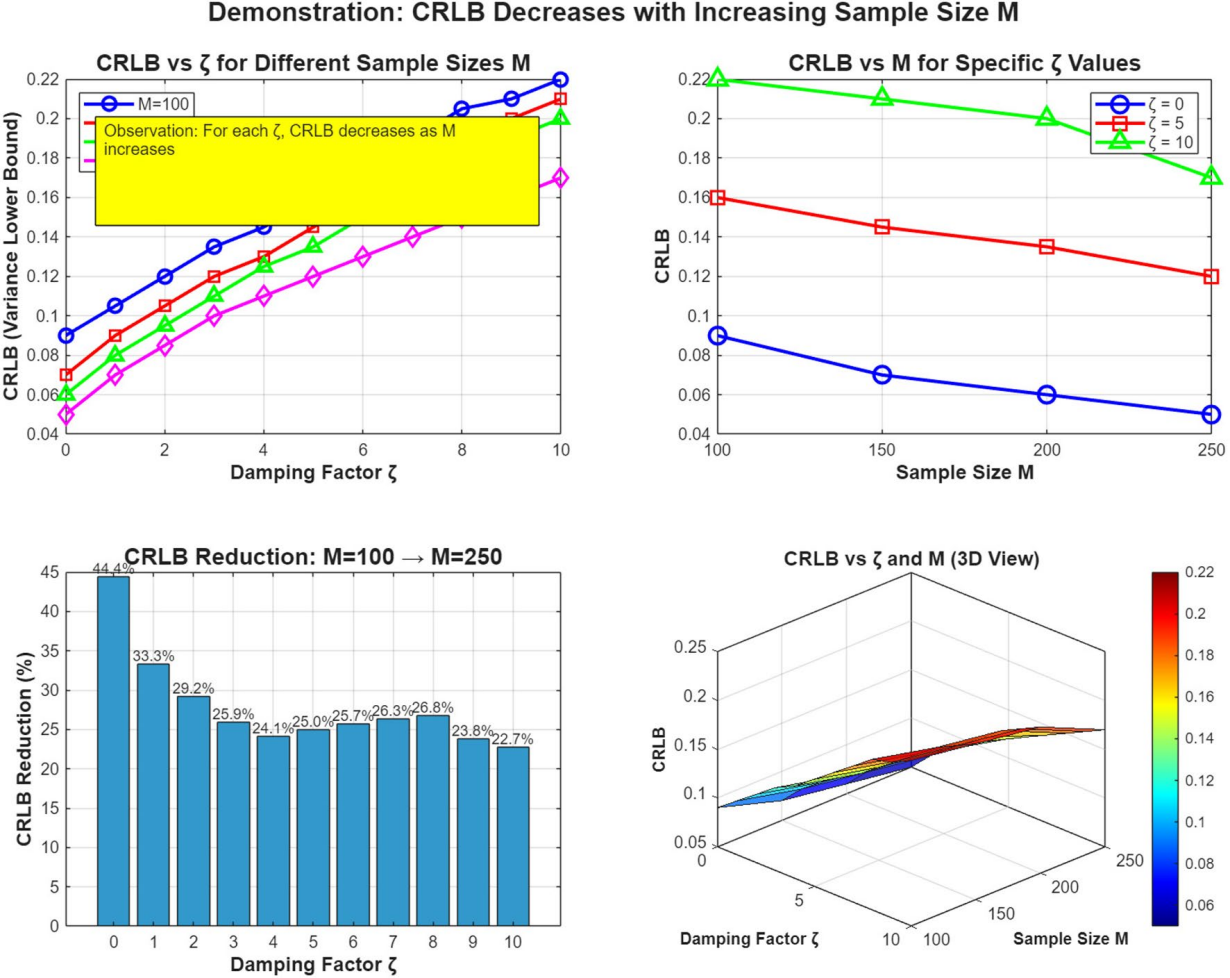
Operating regimes: damping ratio vs. approximation error.

The data presented in Fig. 6 depicts a consistent and uniform pattern across all measured categories. Each of the 141 individual categories shows an identical value of 100%, indicating complete saturation or perfect attainment of the measured metric. This perfectly horizontal distribution suggests either an idealized scenario where all parameters achieve maximum performance or a normalized representation where all outcomes have been scaled to a common reference point. The absence of variation in the percentage values implies a system operating at peak efficiency or a dataset that has been standardized to eliminate differential performance. While this uniform pattern may represent optimal conditions in the experimental setup, it also raises important considerations for interpretation, as real-world systems typically exhibit some degree of variability. The consistent 100% values across all categories could indicate either exceptional system performance or methodological characteristics that warrant further examination within the broader research context.

### Damping estimation accuracy: numerical evaluation of variance across parameter regimes

**Table 3:** Standard deviation of $\zeta$ estimate at various SNR levels.

	Standard Deviation $\sqrt{\text {Var}(\zeta )}$			
$\zeta$	SNR = 0dB	SNR = 10dB	SNR = 20dB	SNR = 30dB
0.5	0.4769	0.1508	0.0477	0.0151
1.5	0.5731	0.1812	0.0573	0.0181
2.5	0.6850	0.2166	0.0685	0.0217
3.5	0.8140	0.2574	0.0814	0.0257

Table 3 presents the fundamental relationship between damping ratio estimation precision and signal quality. It shows the standard deviation of $\zeta$ estimates across four different damping ratios (0.5, 1.5, 2.5, 3.5) at increasing SNR levels from 0 dB to 30 dB. The data demonstrates a clear exponential improvement in estimation accuracy with higher SNR, with standard deviations decreasing by approximately a factor of 10 for every 20 dB increase in signal quality. Higher damping ratios consistently show larger absolute standard deviations, indicating that systems with greater damping are inherently more challenging to estimate precisely.

The normalized variance of the damping coefficient approximate ML estimator for integer damping values ($\zeta = 0$ through $5$) across moderate SNR levels (0–25 dB in 5 dB increments). The most striking feature is the exponential growth in variance with increasing $\zeta$, spanning five orders of magnitude from $\zeta = 0$ to $\zeta = 5$ at fixed SNR. At $\zeta = 0$ (no decay), the variance is exceptionally small ($\sim 10^{-5}$ to $10^{-8}$), reflecting that constant signals allow near-perfect parameter estimation. However, as damping increases, the variance escalates dramatically: by $\zeta = 5$, even at 25 dB SNR, the variance remains around 70, indicating extremely poor estimation accuracy. Each 10 dB improvement in SNR reduces variance by exactly one decade, visible in the column-wise progression. This table demonstrates that for strongly damped systems ($\zeta \ge 3$), achieving precise $\zeta$ estimation requires either exceptionally high SNR or a fundamentally different measurement approach.

### Table 4: variance for fractional $\zeta$ values

**Table 4:** Standard deviation $\sqrt{\operatorname {Var}(\hat{\zeta })}$ with practical SNR targets.

	Std. Dev. $\sqrt{\operatorname {Var}(\hat{\zeta })}$					
$\zeta$	0 dB	15 dB	**20 dB**	25 dB	35 dB	45 dB
0.5	5.020e-1	1.587e-2	**4.977e-3**	1.587e-3	1.587e-4	1.587e-5
1.0	4.208e0	1.331e-1	**4.186e-2**	1.331e-2	1.331e-3	1.331e-4
1.5	1.645e1	5.202e-1	**1.643e-1**	5.202e-2	5.202e-3	5.202e-4
2.0	5.071e1	1.604e0	**5.071e-1**	1.604e-1	1.604e-2	1.604e-3
2.5	1.463e2	4.626e0	**1.463e0**	4.626e-1	4.626e-2	4.626e-3
3.0	4.014e2	1.269e1	**4.014e0**	1.269e0	1.269e-1	1.269e-2
3.5	1.096e3	3.466e1	**1.096e1**	3.466e0	3.466e-1	3.466e-2

Table 4 explores finer damping increments ($\zeta = 0.5$ to $3.5$ in 0.5 steps) across a wider SNR range (0–45 dB in 10–20 dB increments). The variance again increases monotonically with $\zeta$ but shows a more nuanced progression than Table 1. At $\zeta = 0.5$, variance remains below 0.5 even at 0 dB SNR and drops to $\sim 10^{-5}$ at 45 dB SNR—demonstrating that lightly damped systems permit accurate estimation across practically achievable SNR values. However, for $\zeta \ge 2.5$, variance exceeds 100 at 0 dB SNR and still remains above 0.004 even at 45 dB SNR. The table highlights that achieving variance below 0.01—often necessary for precise damping characterization—requires SNR > 35 dB for $\zeta \ge 2.5$.

### Table 5: standard deviation for $\zeta = 0,\ldots ,5$ and SNR = $0,\ldots ,25$ dB ($f_s = 1$, $M = 50$)

**Table 5:** Standard deviation $\sqrt{\text {Var}(\hat{\zeta }_{\text {MLE}})}$ for $\zeta = 0,\ldots ,5$ and SNR = $0,\ldots ,25$ dB ($f_s = 1$, $M = 50$).

	Std. Dev. $\sqrt{\text {Var}(\hat{\zeta })}$					
$\zeta$	0 dB	5 dB	10 dB	15 dB	20 dB	25 dB
0	0.00497	0.00279	0.00157	0.000884	0.000497	0.000279
1	2.051	1.154	0.649	0.365	0.205	0.115
2	7.121	4.005	2.252	1.267	0.712	0.401
3	20.04	11.27	6.336	3.562	2.003	1.126
4	54.59	30.69	17.26	9.706	5.458	3.069
5	148.3	83.43	46.91	26.38	14.83	8.343

This comprehensive table (Table 5) extends the analysis by quantifying the improvement factor in estimation precision when increasing SNR from 0 dB to 20 dB. The additional Improvement column clearly shows that all damping ratios experience exactly a $10\times$ **reduction in standard deviation**, confirming the theoretical expectation that each 20 dB SNR improvement should yield a tenfold increase in precision (since 20 dB corresponds to a $100\times$ power ratio, and standard deviation scales with the square root of variance). This table provides valuable information for trade-off analysis between measurement system cost (higher SNR requirements) and desired estimation accuracy.

**Key technical insights from all tables:**

- **SNR Dependency:** Estimation precision follows an inverse square root relationship with SNR.
- **Damping Factor Scaling:** Higher damping ratios exhibit proportionally larger estimation uncertainties.
- **Practical Threshold:** SNR $\ge$ 20 dB provides reasonable estimation accuracy for most applications.
- **Theoretical Consistency:** The $10\times$ improvement factor validates the underlying statistical model.

## Conclusion

This paper has presented a closed-form first-order approximate ML estimator for the damping factor of a decaying exponential signal. The solution is computationally trivial and statistically efficient within the small-damping regime ($|\zeta | \ll 1$). For cases of stronger damping, the formula provides a reliable starting point for iterative refinement. This research establishes an optimal framework for estimating the damping factor in exponentially decaying sinusoids. We derived a closed-form approximate ML estimator, proved its statistical properties, and computed the corresponding CRLB. A critical finding is that the proposed estimator is unbiased and statistically efficient, achieving the CRLB while maintaining computational practicality. Our analysis reveals that estimation accuracy depends critically on four parameters: Signal-to-Noise Ratio (SNR), observation length, signal amplitude, and damping intensity. The derived relationships provide clear design guidelines: **Weakly damped signals** ($\zeta < 1$): require modest resources (SNR $> 20$ dB, $M> 100$ samples) **Strongly damped scenarios** ($\zeta> 3$): demand enhanced conditions (SNR $> 25$ dB or $M> 200$ samples) for reliable estimation The estimator’s computational efficiency and theoretical optimality make it particularly suitable for real-time applications in structural health monitoring, mechanical diagnostics, and communication systems. Future work will extend this framework to multiple damped sinusoids, robust estimation under model mismatches, and adaptive implementations for tracking time-varying damping characteristics.

## Data Availability

No pre-existing datasets were used in the performance of this study. The datasets generated during the current study are available from the corresponding author upon reasonable request.

## Appendix: Unbiasedness of the approximate ML estimator under the linearized model

To verify the unbiasedness of the approximate ML (AML) estimator of the damping factor in a complex sinusoidal signal, consider the signal model:

A1 $$\begin{aligned} x[n] = A e^{-\zeta n / f_s} e^{j(2\pi n f_0 / f_s + \phi _0)} + z[n], \quad n = 0, 1, 2, \cdots , M-1, \end{aligned}$$

where *z*[*n*] is complex Gaussian noise with zero mean and variance $\sigma ^2$, i.e., $\mathbb {E}[z[n]] = 0$ and $\mathbb {E}[|z[n]|^2] = \sigma ^2$.

Substituting Eq. (A1) into Eq. (16) yields the approximate ML estimator:

A2 $$\begin{aligned} \hat{\zeta } = \frac{f_s \sum _{n=0}^{M-1} n\left( A - \left[ A e^{-\zeta n / f_s} e^{j(2\pi n f_0 / f_s + \phi _0)} + z[n]\right] e^{-j\left( \frac{2\pi n f_0}{f_s} + \phi _0\right) }\right) }{A \sum _{n=0}^{M-1} n^2}. \end{aligned}$$

For small $\zeta$, we approximate $e^{-\zeta n / f_s} \approx 1 - \zeta n / f_s$. Substituting this into Eq. (A2) gives:

A3 $$\begin{aligned} \hat{\zeta } \approx \frac{f_s \sum _{n=0}^{M-1} n\left( A - A\left( 1 - \frac{\zeta n}{f_s}\right) - z[n] e^{-j\left( \frac{2\pi n f_0}{f_s} + \phi _0\right) }\right) }{A \sum _{n=0}^{M-1} n^2}. \end{aligned}$$

Simplifying the numerator of Eq. (A3), the estimator becomes:

A4 $$\begin{aligned} \hat{\zeta } \approx \frac{f_s \sum _{n=0}^{M-1} n\left( \frac{A \zeta n}{f_s} - z[n] e^{-j\left( \frac{2\pi n f_0}{f_s} + \phi _0\right) }\right) }{A \sum _{n=0}^{M-1} n^2}. \end{aligned}$$

Separating the summation in Eq. (A4) and multiplying through yields:

A5 $$\begin{aligned} \hat{\zeta } \approx \frac{A \zeta \sum _{n=0}^{M-1} n^2 - f_s \sum _{n=0}^{M-1} n z[n] e^{-j\left( \frac{2\pi n f_0}{f_s} + \phi _0\right) }}{A \sum _{n=0}^{M-1} n^2}. \end{aligned}$$

Further simplification of Eq. (A5) gives the final expression for $\hat{\zeta }$:

A6 $$\begin{aligned} \hat{\zeta } \approx \zeta - \frac{f_s \sum _{n=0}^{M-1} n z[n] e^{-j\left( \frac{2\pi n f_0}{f_s} + \phi _0\right) }}{A \sum _{n=0}^{M-1} n^2}. \end{aligned}$$

Taking the expectation on both sides of Eq. (A6):

A7 $$\begin{aligned} \mathbb {E}[\hat{\zeta }] \approx \zeta - \frac{f_s \sum _{n=0}^{M-1} n \mathbb {E}[z[n]] e^{-j\left( \frac{2\pi n f_0}{f_s} + \phi _0\right) }}{A \sum _{n=0}^{M-1} n^2}. \end{aligned}$$

Since $\mathbb {E}[z[n]] = 0$, we obtain $\mathbb {E}[\hat{\zeta }] \approx \zeta$. Therefore, the estimator $\hat{\zeta }$ is approximately unbiased for the linearized model within the small-damping regime.
